# Social interactions within the Sudanese healthcare system: traditional healers and psychiatrists

**DOI:** 10.1192/bji.2022.27

**Published:** 2023-05

**Authors:** Randa Ahmed Abdalrheem Altamih, Osman Kamal Osman Elmahi

**Affiliations:** 1 Researcher, Faculty of Medicine, University of Khartoum, Khartoum, Sudan; 2 Researcher, Faculty of Medicine, Ibn Sina University, Khartoum, Sudan. Email: osman19091995@gmail.com

**Keywords:** History of psychiatry, primary care, Sudan, traditional psychiatry, community mental health teams

## Abstract

From a cultural perspective, traditional healing has had a substantial impact on psychiatric management in rural African communities, but the services provided by traditional healers are not integrated with the mental health services provided by primary healthcare. In Sudan, modern psychiatry has seen minimal development beyond the capital city of Khartoum. In rural communities, traditional health practitioners (THPs) are the first point of entry to mental health services. Effective collaboration between THPs and consultant psychiatrists should be encouraged by the introduction of health education that targets THPs, especially in rural communities. This would facilitate the integration of mental health services into primary healthcare and help achieve universal health coverage for psychiatric disorders in Sudan.

Cultural practices in the management of psychiatric conditions play a very important role in African communities through the services of traditional health practitioners (THPs).^1^ Federal health ministries and other regulatory bodies provide mental health services across the globe, but cultural practices in psychiatric management have received little credit from higher institutions, despite the important role played by THPs in rural communities. According to the World Health Organization (WHO), approximately 80% of rural populations in low-income and lower middle-income countries are dependent on THPs for their health needs.^2^ Sudan is a nation in which mental health services provided by THPs are highly respected. Rural communities perceive THPs as the first point of entry into treatment for psychiatric diseases. Also, THPs are preferred by rural communities because of their accessibility and affordability. The treatment of severe psychiatric conditions is not provided by primary care settings, and THPs are therefore preferred by the rural population in Sudan. Despite the fact that the WHO recognises the potential value of integrating mental health services into primary healthcare through the Traditional Medicine Programme, primary healthcare centres in Sudan have yet to provide psychiatric care facilities.^2^

## Current picture of interaction between traditional healers and psychiatrists

In Sudan, traditional healers play a key role in the provision of mental health services, despite the absence of regulations concerning their services. The WHO has strongly advocated collaboration between THPs and primary care physicians since the 1970s, in conjunction with the integration of THPs into national health systems. This integration would lead to improvement in the care provided through regular evaluation of the utility, effectiveness and safety of care delivery by THPs.

Traditional healthcare practices in Sudan have similarities to those in Kenya. For example, counselling rituals have been applied to treat depression. Faith-based healers adopt prayer-based practices and cast out demons to treat neurotic and psychotic symptoms.^3^ If an attempt at integration with conventional medical services were made, evaluations would allow improved understanding of THP practices and mitigation of any associated risks, coupled with the ability to determine where safe traditional practices could be implemented by national healthcare systems.^4^

A small majority of psychiatry consultants and residents in Khartoum believed in the value of collaborating with THPs, and 58% were willing to support the integration of traditional healers in patient-related practices and in influencing stakeholders.^5^ Despite traditional healing not being an evidence-based practice, it was found throughout Sudan that psychotic symptoms declined among patients attending traditional healing centres,^6^ thus indicating the possible presence of a role for THPs in delivery of mental health services. Furthermore, it was established that a sizeable minority of psychiatrists in Sudan (43%) agreed with the practice of *rogia* – a form of hypnotism similar to that used by psychologists developing therapeutic alliances – as a means of relieving neurotic symptoms.^5^ The practice of *rogia* is a form of faith-based counselling performed in rural Sudan by the local caliphate, in which Quranic verses are used to treat depression, anxiety and demonic thoughts and persecutions experienced by individuals. These practices are more readily available in rural Sudanese communities with a caliphate style of authority, whereby a caliph presides over the community.

## Recommendations

In rural and remote areas in Sudan, THPs are the first points of access to mental healthcare because there is an extremely low coverage of mental health services.^6^ At the level of rural communities, basic training programmes, including written communication and basic information technology skills, to enable THPs to escalate to psychiatric care facilities patients whose symptoms are refractory to herbal treatment would yield a considerably stronger interaction between THPs and consultant psychiatrists. This would be facilitated further by the prioritisation of mental health services by the Federal Ministry of Health in Sudan. There should be integration into primary healthcare and subsequent ease of access to mental health services for both THPs and their patients in both urban and rural communities. Elements of integration of mental health services into universal health coverage would necessitate long-term planning, a community-based care package and a compassionate stance to encourage patient and community engagement. Achievement of this integration could begin through health education of rural communities by THPs about the importance of modern psychiatry in treating psychosocial disorders.^7^

Budgeting and stigma are common barriers faced by people with mental disorders in Sudan. These barriers could be combated by providing mental health education training to THPs from established psychiatrists and general practitioners. The trained THPs could then work in collaboration with these experts, delivering their services to rural communities. This proposed way of delivering services would adopt a patient-centred approach and would go some way towards achieving universal health coverage at minimal expense.^8^

## Conclusions

In Sudan, there has been minimal development of psychiatric services since their introduction to the country in the mid-20th century. THPs are the main source of treatment for psychiatric conditions in rural communities. The education of THPs in rural communities by mental health experts could be a productive collaboration. They could work together with the aim of achieving universal health coverage, each providing crucial elements to achieve a productive interaction between cultural practitioners and consultant psychiatrists.

## Data Availability

Data availability is not applicable to this article as no new data were created or analysed in this study.
